# HLA-*DRB1* Class II antigen level alleles are associated with persistent HPV infection in Mexican women; a pilot study

**DOI:** 10.1186/1750-9378-8-31

**Published:** 2013-08-30

**Authors:** Sofía Bernal-Silva, Julio Granados, Clara Gorodezky, Carmen Aláez, Hilario Flores-Aguilar, Ricardo M Cerda-Flores, Geraldina Guerrero-González, Lezmes D Valdez-Chapa, José Morales-Casas, Juan Francisco González-Guerrero, Hugo A Barrera-Saldaña

**Affiliations:** 1 Department of Biochemistry and Molecular Medicine, School of Medicine, Universidad Autonoma de Nuevo Leon, Av. Madero ote. s/n esq. Eduardo Aguirre Pequeño, Col. Mitras Centro, CP 64460, Monterrey, Nuevo Leon, Mexico; 2 Department of Transplantation. Instituto Nacional de Ciencias Medicas y Nutricion Salvador Zubirán, Tlalpan, D.F., Mexico; 3 Department of Immunology & Immunogenetics. Instituto de Diagnostico y Referencia Epidemiologicos. Secretary of Health, Tlalpan, D.F, Mexico; 4 School of Nursery, Universidad Autonoma de Nuevo Leon, Eduardo Aguirre Pequeño s/n, Col. Mitras Centro, Monterrey, Nuevo Leon CP 64460, Mexico; 5 Department of Gynecology and Obstetrics, Hospital Universitario. Universidad Autonoma de Nuevo Leon, Av. Gonzalitos s/n, Col Mitras Centro, Monterrey, Nuevo Leon CP 64460, Mexico; 6 Centro Universitario Contra el Cancer, Universidad Autonoma de Nuevo Leon, Av. Gonzalitos s/n, Col Mitras Centro, Monterrey, Nuevo Leon CP 64460, Mexico; 7 Department of Cytology, Centro Universitario de Salud, Universidad Autonoma de Nuevo Leon, Av. Gonzalitos s/n, Col Mitras Centro, Monterrey, Nuevo Leon CP 64460, Mexico

**Keywords:** HPV, HLA class II, Susceptibility, Persistent infection, *DRB1*

## Abstract

**Background:**

Persistent infection with high-risk human papillomavirus (HPV) is a major risk factor for malignant lesions and cervical cancer. A widely studied element in the search for genetic factors influencing risk HPV infection diseases is allelic variation of the human leukocyte antigen (HLA) locus. The study was designed to search for HLA susceptibility alleles contributing to the persistence of HPV infection in Mexican women.

**Methods:**

A total of 172 subjects were divided into three groups: 1) HPV–persistent patients; 2) HPV–cleared; and 3) HPV–reinfected patients. They were screened for HPV types using a polymerase chain reaction (PCR). PCR-sequence specific oligonucleotide probes (PCR-SSOP) was used for HLA *DRB1* and *DQB*1 typing.

**Results:**

We observed that HLA-DQB1*0501 allele might be associated with susceptibility of reinfection with HPV (p = 0.01, OR = 4.9, CI 95% = 1.3 -18.7). Allele frequency of HLA-DRB1*14 was particularly reduced in patients with cancer when compared with the HPV–persistent group (p = 0.04), suggesting that this allele is a possible protective factor for the development of cervical cancer (OR = 2.98). HLA-DRB1*07 might be associated with viral clearance (p = 0.04).

**Conclusions:**

Genetic markers for HPV infection susceptibility are different in each population, in Mexicans several HLA-*DQB1* alleles might be associated with an enhanced risk for viral persistence. In contrast, DRB1*14, seems to confer protection against cervical cancer.

## Background

Infection by human papillomavirus (HPV) is a common sexually transmitted infectious disease and most sexually active women have been infected during their lifetime [1]. Persistent infection by oncogenic HPV is a risk factor for developing cervical cancer (CC), but only a small number of infected women develop precancerous lesions, and even fewer develop invasive cancer. This indicates that other risk factors, such as genetic predisposition, are also very important. The most important protective immune mechanism for CC is the suppression or elimination of HPV infection by the cell mediated immune response (CMR) within 1–2 year of exposure [2]. A number of genetic risk factors have been identified, but their effects are generally weak. The most prominent among the known risk factors is the human leukocyte antigen (HLA) complex, which plays a critical role in susceptibility to CC [3]. Since the first reported association of HLA-DQ3 with CC [4], a large number of studies of HLA association with CC have been published with variable results depending on the ethnic group.

Affected sib-pair analyses in Swedish women, revealed an excess sharing of *DPB1*, *DQB1* and *DRB1* alleles, with the strongest evidence for *DQB1* and *DRB1 *[5]. An analysis of the entire HLA region using microsatellites, confirmed the association of DQ/DR genes with the risk of developing CC [6]. An increased risk of CC was also observed in homozygote carriers of the MICA allele 184, suggesting a recessive effect of this locus [6]. DRB1*04:07-DQB1*03:02 and DRB1*15:01-DQB1*06:02 were clearly associated with susceptibility to HPV-16 positive invasive CC [7], high squamous intraepithelial lesion (HSIL), and carcinoma in situ, but only in HPV-16 infected patients [7]. DRB1*15–DQB1*06:02 haplotype was also found increased in CC and CC-HPV-16 positive Brazilian patients [8]. Part of the herein authors published an association of, HLA-DQA1*03:01 in Honduran women [9], which is in linkage disequilibrium with all HLA-DR4 subtypes in Mestizos, as an increased risk of developing HSIL and CC. The DQB1*03 group, or some of DQ3 alleles, have been linked with CC susceptibility in different European, Asian, and African-American populations [7,10-13]. Some DR-DQ haplotypes containing DQB1*03:01 allele have been positively associated with CC susceptibility: DRB1*11:01-DQB1*03:01 in Senegalese [14] and US white Europeans [15] and DRB1*04:01-DQB1*03:01 in US white Europeans [15] and British females [16]. DRB1*11:02-DQB1*03:01 was also increased in Hispanics with carcinoma *in situ* or HSIL [7]. In contrast, there are studies in Canadian and Senegalese women in which it was not possible to confirm an association between the DQB1*03 allele and the risk of CC or high grade cervical intraepithelial neoplasia [14,17] and some studies find a protective role of this allele against HPV infections and CC [18-20].

Protection has been mainly linked with the HLA-DRB1*13 group: DRB1*13:01 in patients from Costa Rica [21], and DRB1*13:01-DQB1*06:03-DQA1*01:03 in Swedish [22], French and Dutch women with CC [23]. A protective effect against CC progression was also claimed to be correlated with DQB1*05, DQA1*01:01/04, DRB1*01:01 and DRB1*13:02 in Brazilians [8]. In Caucasians, HLA-DRB1*13 and HPV-16/18-negative status, were independently associated with an increased probability of regression of low squamous intraepithelial lesion (LSIL), also suggesting a protective effect against CC progression [24].

Several HLA and CC association studies have been published in Mexican women. A significant increase of HLA-A2 and HLA-DR5 and a significant decrease of HLA-DR6, HLA-DR2 and HLA-DR1 were shown in women with CC, using serological techniques [25]. The DRB1*15-DQB1*06:02 haplotype was associated with CC in HPV-16 positive females and several DR4-DQB1*03:02 and DQB1*03:01 haplotypes were negatively associated with CC. However, all alleles except for those belonging to the DQB1 locus were performed at the antigen level [26]. Finally, a recent report looking for the correlation of HPV-16 and CC in Mexican women, showed that DRB1*04:03 influences the risk of developing HSIL, independently of HPV16 and DRB1*01:01, it may confer protection against the development of CC. The HLA-DRB1*04:07 allele was increased in HPV16 positive patients, confirming that the HLA association is HPV dependent [27].

The aim of the present study was to investigate if HLA class II alleles are associated with persistence of infection, independently of its progression to CC. We observed that HLA-DQB1*0501 allele might be associated with susceptibility of reinfection with HPV and HLA-DRB1*14 seems to confer protection against CC.

## Results and discussion

This study is part of an open population project to investigate asymptomatic women with HPV infection in 2002 (ML Garza-Rodríguez and HA Barrera-Saldaña, unpublished results). One of the main objectives of this work was to do a follow up of these women to determine the possible role of HLA class II genes in the fate of the viral infection. Although it is known that class I genes are very important in the immune response because of their involvement in antigen presentation of viral peptides on the surface of immune system cells, it is also known that the presentation of these peptides by class II alleles play a crucial role in protection by CD4+ lymphocytes.

All patients included, were HPV positive at the beginning of the investigation in 2002. The group (n = 172) was divided into three subgroups during their follow-up in 2005 as follows: 1) HPV-persistent (n = 21), composed of women who had a persistent viral infection, meaning infection with the same HPV-type as in 2002 during their second visit (2005); 2) HPV-cleared group (n =138), formed by women who eliminated the viral infection by their second visit, evidenced by negative molecular detection; and 3) HPV-reinfected (n = 13), that consisted of women who eliminated the infection but subsequently acquired a new infection with a different HPV-type, evidenced by molecular detection in the cervical sample in 2005. No patient had developed CC by the second visit of the study.

The mean age of the 172 women included in the analyses was 40.3 years (SD ± 11.2 years). All were residents of the city of Monterrey, capital of the state of Nuevo Leon, but only 55.9% were born in the state. The remaining 44.1% averaged 30.4 years (SD ± 11.9 years) (40.2%) living there with most being natives from the neighboring state of San Luis Potosí (Table 1). At the second visit, 34 women (19.7%) were positive for HPV infection of which eight (23.5%) harbored co-infection with multiple HPV types. The analysis showed that 61.5% of infections were caused by high-risk HPV types (HR-HPV), 30.8% by low-risk types (LR-HPV) and 7.7% by unknown oncogenic risk type viruses (Table 1). The most prevalent viral types were: HPV-83 (38.1%), HPV-62 (23.8%) and HPV-18 (19%), followed by HPV-39, -52, -61 and −84 (14.3% each) and for HPV-16, -51, 53 and 81 (9.5% each). Only 21 patients (12.2%) remained infected with the same viral type (persistent infection), compared with the results of the first visit in 2002 (Table 1).

**Table 1:** Characteristics of the study population and most prevalent HPV types

	**N**	**%**
Study group	172	100
Age		
Monterrey as city of residence	172	100
Monterrey, N.L. as birth city	96	55.9
City of birth different from Monterrey	76	44.1
HPV positive at 2002	172	100
HPV positive for any type at 2005	34	19.7
HPV Co-infections	8	23.5
HR-HPV		61.5
LR-HPV		30.8
Unknown oncogenic risk type viruses		7.7
HPV positive at 2005 but with the same type as in 2002*	21	12.2
HPV-cleared at 2002	138	80.2
Most prevalent HPV types	Type	%
	−83	38.1
	−62	23.8
	−18	19
	−39, 52, 61, 84	14.3 (each)
	−16, 51, 53, 81	9.5 (each)

*Persistent infection.

Patients in the viral persistence group were considered cases to be contrasted against the other two groups (HPV-cleared and HPV-reinfected), as well as against a group of healthy Mexican women with a normal Papanicolaou smear (considered healthy individual controls, n = 88) described elsewhere [27], and against a group of Mexican women with CC (considered disease controls, n = 104) described elsewhere as well [26] (both were considered external controls). Since viral persistence has been directly associated with the development of premalignant lesions and CC, in order to search for possible associations of alleles with susceptibility or resistance to viral persistence and CC, we decided to compare our three study groups with the previously mentioned external control groups [26,27].

### HLA-*DQB1* alleles

HLA class II gene *DQB1* genotyping analysis was conducted in all 172 at the high resolution level. All alleles were in Hardy-Weinberg equilibrium. The most prevalent alleles were DQB1*03:02 [gene frequency (g.f.) = 0.235], DQB1*03:01 (g.f. 0.218), DQB1*04:02 (g.f. 0.154), DQB1*02:01 (g.f. 0.122) and DQB1*05:01 (g.f. 0.078) (Table 2). When comparing the g.f. of alleles found in the HPV-reinfected group against those of the HPV-cleared group, we found that DQB1*05:01 was associated with susceptibility to reinfection (p = 0.01, OR = 4.9, CI 95% = 1.3 -18.7) (data not shown). No other significant associations were observed; it was not possible to confirm the protective effect against progression of the disease observed in Brazilians [8].

**Table 2:** ***DQB1 *allele gene frequencies (g.f.) in the studied groups**

	**HPV-Persistent**		**HPV-cleared**		**HPV-reinfected**		**Total**	
	N* = 42		N* = 276		N* = 26		**N = 344**	
*DQB1*	n	g.f.	n	g.f.	n	g.f.	n	g.f.
03:02	9	0.214	70	0.254	2	0.077	81	0.235
03:01	11	0.262	59	0.214	5	0.192	75	0.218
04:02	6	0.143	44	0.159	3	0.115	53	0.154
02:01	3	0.071	34	0.123	5	0.192	42	0.122
05:01	6	0.143	15	0.054	6	0.231	27	0.078
06:09	1	0.024	2	0.007	0	0.000	3	0.071
06:02	1	0.024	15	0.054	1	0.038	17	0.049
05:03	1	0.024	7	0.025	1	0.038	9	0.026
06:04	0	0.000	8	0.029	0	0.000	8	0.023
06:03	1	0.024	5	0.018	1	0.038	7	0.020
03:03	1	0.024	3	0.011	1	0.038	5	0.015
05:02	0	0.000	4	0.014	1	0.038	5	0.015
06:01	0	0.000	4	0.014	0	0.000	4	0.012
03:16	1	0.024	1	0.004	0	0.000	2	0.006
03:04	0	0.000	2	0.007	0	0.000	2	0.006
03:12	1	0.024	0	0.000	0	0.000	1	0.003
06:22	0	0.000	1	0.004	0	0.000	1	0.003
06:14	0	0.000	1	0.004	0	0.000	1	0.003
03:05	0	0.000	1	0.004	0	0.000	1	0.003

*N = number of alleles (n × 2).

### HLA-DRB1 alleles

For the analysis of the HLA-*DRB1* locus, 95 patients were included. We also divided these patients into the three categories described above: 1) HPV-persistent group, n = 21; 2) HPV-cleared, n = 61; and 3) HPV-reinfected, n = 13. We calculated the number of subjects required in the comparison group (HPV-cleared group) to corroborate the presence of a significant difference; this calculation indicated that a 1:3 ratio would be sufficient for most comparisons. Base on this, and taking into consideration the high cost to genotype HLA genes, we decided to study 61 subjects in the HPV-cleared group. These subjects were randomly selected from the original group (n = 138).

The five alleles more frequently found in the HPV-persistent group were: HLA-DRB1*04 (g.f. = 0.262), DRB1*14 (g.f. = 0.143), DRB1*08 (g.f. = 0.119), DRB1*01 (g.f. = 0.095) and DRB1*11 (g.f. = 0.095). For the HPV-cleared patients the most frequent alleles were HLA-DRB1*04, DRB1*08, DRB1*07, DRB1*14 and DRB1*11, with a g.f. of 0.262, 0.180, 0.107, 0.082 and 0.074, respectively. The most common alleles found in the HPV-reinfected group was HLA-DRB1*08, followed by DRB1*07, DRB1*14, DRB1*01 and DRB1*03. All the g.f. described above and those of the remaining studied alleles found in the three groups and in the external control groups, are shown in Table 3.

**Table 3:** ***DRB1 *allele gene frequencies (g.f.) in the studied groups**

	**Healthy individuals**^**26**^		**HPV-Persistent**		**HPV-cleared**		**HPV-reinfected**		**Cervical cancer**^**25**^	
	N* = 176		N* = 42		N* = 122		N* = 26		N* = 208	
***DRB1***	**n**	**g.f.**	**n**	**g.f.**	**n**	**g.f.**	**n**	**g.f.**	**n**	**g.f.**
04	49	0.278	11	0.262	32	0.262	1	0.038	63	0.3029
08	29	0.164	5	0.119	22	0.180	4	0.154	41	0.1971
07	7	0.039	2	0.048	13	0.107	4	0.154	11	0.0529
14	16	0.090	6	0.143	10	0.082	3	0.115	11	0.0529
11	11	0.062	4	0.095	9	0.074	2	0.077	8	0.0385
15	13	0.073	1	0.024	5	0.041	1	0.038	12	0.0577
03	9	0.051	1	0.024	8	0.066	3	0.115	13	0.0625
01	15	0.085	4	0.095	7	0.057	3	0.115	12	0.0577
13	13	0.073	3	0.071	9	0.074	1	0.038	16	0.0769
16	9	0.051	2	0.048	5	0.041	1	0.038	13	0.0625
09	2	0.011	1	0.024	1	0.008	0	0.000	0	0.0000
12	2	0.011	0	0.000	1	0.008	1	0.038	1	0.0048
10	1	0.005	2	0.048	0	0.000	2	0.077	7	0.0336

*N = number of alleles (n × 2).

The results for the healthy individual external control group [27], and those for the external disease control group [26], respectively, are described in the first and last columns of Table 3. The most frequent alleles of the former group were HLA-DRB1*04, DRB1*08 and DRB1*14 with the first two being also the most frequent in the latter group.

DRB1*04 was found decreased in patients in the HPV-reinfected group (Table 4) and this difference was statistically significant when compared with the patients in the HPV-persistent group (p = 0.02, OR = 8.8, CI 95% = 1.0-196; Table 4) but no significant difference was found in comparisons made against the rest of the groups (data not shown). However, the DRB1*04 allele has been associated with susceptibility to HSIL and CC development in different Caucasian and Mexicans patients [27-29]. This finding suggests that the absence of DRB1*04 is probably associated with an increased risk of reinfection by different HPV genotypes. Although it is difficult to draw this conclusion because the small number of patients carrying the allele in the HPV-reinfected group (only one, Table 3).

**Table 4:** Odds ratio of the HLA-DRB1 alleles found associated in the studied groups

**Control group**	**Case group**	**Class II allele**	**p value, OR and CI 95%**
HPV-reinfected	HPV-persistent	DRB1*04	0.02, OR 8.8 (1.0 -196)
Healthy individuals	HPV-cleared	DRB1*07	0.04, OR 2.8 (1.03 -8.3)
Healthy individuals	HPV-persistent + reinfected + cleared	DRB1*07	0.04, OR 2.7 (1.03 -7.2)
HPV-cleared	HPV-persistent + cervical cancer	DRB1*07	0.05, OR 0.45 (0.20-1.02) ^n.s.^
HPV-cleared + HPV-reinfected	HPV-persistent + cervical cancer	DRB1*07	0.03, OR 0.42 (0.19-0.89)
Healthy individuals	HPV-persistent + cervical cancer	DRB1*07	0.01, OR 0.31 (0.12-0.79)
Healthy individuals	HPV-persistent + reinfected	DRB1*10	0.03, OR 10.9 (1.12 -261)
Healthy individuals	HPV-reinfected	DRB1*10	0.04, OR 14.5 (1.27-167)
Healthy individuals	HPV-persistent + cervical cancer	DRB1*10	0.05, OR 6.53 (0.82-52) ^n.s.^
Cervical cancer	HPV-persistent	DRB1*14	0.07, OR 0.34 (0.11 -1.09) ^n.s.^

^n.s.^ No significant.

On the other hand, when comparing the HPV-cleared group against the external healthy individuals group [27] in order to explore the role of the HLA gene in viral clearance, an increase in the g.f. of the DRB1*07 was observed in the former group (p = 0.04, OR = 2.88, CI 95% = 1.03 – 8.3; Table 4). We also found the same increase in the OR for that allele when pooling the three groups (HPV-persistent + HPV-cleared + HPV-reinfected) and comparing them against the external healthy individuals group (p = 0.04, OR = 2.7, CI 95% = 1.03-7.2; Table 4). The three groups that were pooled for this analysis have in common the fact that all patients were infected with HPV and this was proved with the results of the 2002 genotyping results. Thus, the results suggest a probable association between this allele and the risk of being infected with HPV at some time in life. However, when we analyzed the HPV-cleared group and the HPV-reinfected group together (HPV-cleared + HPV-reinfected) and compared them against a group formed by the HPV-persistent group and cervical cancer external group (given that subjects in the cervical cancer group must have had an HPV persistent infection for cervical cancer development) a protective role of this allele against viral persistence was found (p = 0.03, OR = 0.42, IC 95% = 0.19-0.89; Table 4). So, it was thought that what was seen was the protective role of this allele against viral persistence but the association with risk of being infected in some point of the life. To verify the protector role of this allele against a persistent infection, an analysis comparing the healthy individuals group against viral persistence group (HPV-persistent + Cervical cancer groups) was conducted. With this analysis it was possible to confirm (p = 0.01, OR = 0.31, IC 95% = 0.12-0.79) the results about the protective role of DRB1*07 allele. This suggests that the DRB1*07 allele may protect against viral persistence and consequently, individuals carrying this allele, efficiently eliminate HPV infection. It is important to mention and take into account that the present study is an exploratory work which should be confirmed in a larger number of samples.

Comparing the g.f. of alleles found by grouping the HPV-persistent and the HPV-reinfected groups and comparing them against the external healthy individuals group [27] an elevated OR value for the DRB1*10 allele (p = 0.03, OR 10.9, CI 95% = 1.12 to 261,) was detected (Table 4). The elevated value of the OR for the DRB1*10 allele (Table 4), showed a possible association between this allele and the risk of being infected with HPV at some point in life. There are no reports on the association of this allele with CC or HPV infection. The only reports found in the literature are about its association with diseases such as tuberculosis and rheumatoid arthritis in others populations [30,31]. No association was found when the three groups were pooled (HPV-persistent + reinfected + cleared) and compared against the healthy individuals group (data not shown). It was not possible to make comparisons using the HPV-cleared group alone, because there was not any patient with DRB1*10 allele expression in that group. The only association found for this allele was when the HPV-reinfected group was compared against the healthy individuals group (p = 0.04, OR = 14.5, IC 95% = 1.27-167). Nonetheless, the clinical relevance of this finding about the possible role of this allele in protection against an HPV infection needs to be explore with a higher number of patients since the gene frequency of this allele was low in the study population and no patient in the HPV-cleared group was carrying the DRB1*10 allele (Table 3).

Interestingly, the DRB1*14 allele showed a decreased OR value of 0.34 (CI 95% = 0.11 to 1.09), when the latter two groups were compared (Table 4). HLA-DRB1*14 allele is possibly associated with protection for developing CC in patients with viral persistence. This is the first report of the protective role of this allele against CC in Mexican women. In a previous similar study from our population [26], it was found that particularly the DRB1*15 allele was associated with a risk for CC and no allele was associated with protection. These same authors did not find any significant result for the DRB1*14 allele. However, their study design was different from that of the present work. Indeed, while the present study focused on HPV infection and persistence in a cohort followed for three years, in the referred study, the authors focused on confirmed cases of CC. Therefore, it agrees with our finding of an association between the DRB1*14 allele and a low risk of developing CC in women with viral persistence. In another relevant study, Brazilian women from Sao Paulo, with persistent HPV-infection, showed an association between HLA class II alleles and natural history of CC [8]. Some of the immunogenic peptides presented by class I alleles could be part of larger peptides that are presented through class II alleles. This could be the case for the DRB1*14 allele that when presenting certain peptides may help in preventing persistent infections from evolving to CC.

Although the number of patients in the groups was too low to allow any statistical comparisons between the different haplotypes and genotypes formed with the DRB1*14, DRB1*04, DRB1*07 and DRB1*03 alleles, it is possible that other variants of the HLA-*DR* gene could be contributing risk to viral persistence as haplotypes and even as genotypes. This data could be strengthened by the characterization of allelic subtypes (which until 2010, more than 90 molecular variants have been described). Likewise, the genotype for the patient in the HPV-reinfected group carrying the DRB1*04 allele (Table 3) is DRB1*04/DRB1*08 (data not shown) and a comparison was made with the HPV-persistent group, in which no patient was positive for the genotype previously mentioned (data not shown), although five patients have the DRB1*04 allele in this group (Table 3, HPV-persistent group, n =11, g.f. = 0.262) (OR = 18, CI 95% = 0.5 to 3.6) (data not shown). The information above suggest that these individuals probably clear the viral infection in an efficiently manner. This similarly could be the case for the genotypes of the DRB1*07 allele, having found two patients carrying this allele in the HPV-persistent group under the genotypes DRB1*07/DRB1*14 and DRB1*07/DRB1*01 (data not shown). This other finding is interesting because in the HPV-cleared group, eleven alleles were DRB1*07 and none were part of the mentioned genotypes. These data support the notion that DRB1*07 positive individuals are good fighters of viral infection.

The protective role of the DQB1*03:01 allele has been described in other populations [18-20]. Our study did not confirm this association. Nevertheless, it is known that in Mexicans there is a strong linkage disequilibrium between this allele and the HLA-DRB1*04 allele [32]. Interestingly, in our study the aforementioned allele was associated with a lower probability of evolving to CC. Therefore, we consider it important to conduct an extensive analysis of both class II genes to discern what precise role they play in the presentation of peptides by DRB1*04.

We attempted to make a comparison of haplotypes and genotypes among groups in order to check if *DQB1* alleles in the haplotypes were different, but the number of patients was too low.

## Conclusions

Our study suggests that it is necessary to identify genetic markers of HPV infection susceptibility for each population, since the distribution differs in distinct ethnic groups. It also supports the notion that women who do not adequately present the viral antigens, may require comprehensive vaccination, while those with certain protective alleles might require a different preventive approach. Such is the case of women positive for DRB1*14 or DRB1*07. It is evident that to adopt effective preventive approaches, it is be extremely helpful to take into account the immunogenetic profile of the population at risk, which in turn will be important given the variation in the magnitude of CC risk in each unique ethnic group.

## Methods

### Patients

The patients included in this study were recruited from a group of 600 HPV infected women. Detection was done using a polymerase chain reaction (PCR) in a prevalence study that included a total of 3,082 women in 2002 (ML Garza-Rodríguez and HA Barrera-Saldaña, unpublished results). In total, 188 patients were recruited during 2005–2006. Sixteen were excluded because of insufficient sample or due to pregnancy; thus, 172 women were included. The sample was divided into three groups: 1) HPV-persistent group: patients with the same HPV-type as in 2002 (n = 21); 2) HPV-cleared group: patients that cleared their HPV infection (n = 138); 3) HPV-reinfected group: patients with subsequent infections by an HPV-type different than that of 2002 (n = 13). Participants were fully informed of the aim of the research project through informative sessions. This study was a case–control analysis and all patients read and signed an informed consent. The protocol was approved by the Research and Ethics Committee of all participant Institutions and have been performed in accordance with the ethical standards laid down in the 1964 Declaration of Helsinki and its later amendments.

### Cervical sampling and HPV detection

Cervical scrapes for HPV testing were taken using a cervical brush (Colpotre® Chico, CA) and collected in liquid cytology medium. HPV detection was performed by PCR amplification performed with the PGMY 09/11 primers system, followed by viral type discrimination using reverse line blot (Roche Molecular Diagnostics, Inc., Alameda, CA). PCR was carried out according previous methods [33,34]. HPV typing was performed by reverse hybridization of the PCR product to oligonucleotide probes for 37 HPV genotypes attached to a nylon strip using colorimetric identification. Interpretation was done according to the manufacturer’s insert.

### HLA-*DQB1* genotyping

Polymorphic variants within the second exon of *DQB1* were detected by a reverse Dot-blot technique with the INNO-Lipa HLA typing kits (Innogenetics, Inc., Gent, Belgium). The interpretation was done using software provided by the manufacturer.

### HLA-*DRB1* genotyping

The HLA-*DRB1* alleles were detected with a PCR-Luminex typing method using the Lifecodes HLA-SSO class II typing kit (Tepnel-Gen-Probe Transplant Diagnostics, Inc., Stamford, CT). DNA was PCR amplified with biotinylated primers and the PCR products were denatured and hybridized to the complementary oligonucleotide probes attached to the microspheres designed for the Luminex Instrument. The Luminex Instrument is able to quantify the relative amounts of labeled PCR product hybridizing to each Luminex microsphere and types the probes as having positive or negative reactivity with the amplified DNA sample. Genotype determination and data analysis were performed automatically, using the software provided with the kit.

### Statistical analyses

The observed genotype frequencies in the controls were tested for Hardy-Weinberg equilibrium. The HLA frequencies of *DRB1* and *DQB1* alleles were compared between the groups using the two-sided Fisher exact test with 2 × 2 contingency tables or, where appropriate, by the *χ*^2^ test with Mantel-Haenszel correction; the p values of < 0.05 were considered significant. The Bonferroni correction for multiple tests was not required. Haldane’s odds ratios (OR) within the 95% confidence interval (CI) were calculated.

## Abbreviations

HPV: Human papillomavirus; CC: Cervical cancer; PCR: Polymerase chain reaction; PCR-SSOP: Sequence specific oligonucleotide probes PCR; CMR: Cell mediated immune response; HLA: Human leukocyte antigen; HSIL: High squamous intraepithelial lesion; LSIL: Low squamous intraepithelial lesion; HR-HPV: High-risk HPV types; LR-HPV: Low-risk types; g.f: Gene frequency; OR: Odds ratios; CI: Confidence interval.

## Competing interests

There are no financial competing interests (political, personal, religious, ideological, academic, intellectual, commercial or any other) to declare in relation to this manuscript.

## Authors’ contributions

Conception and design of experiments: BSHA. Experiments were performed by: BSS. Data analysis: BSS, AC, GC, FAH, CFRM, GJ. Contributed reagents/materials/analysis tools: BSHA, VChLD, GGJF, MCJ, GGG, CFRM, GJ. Drafted the paper: BSS, GJ, GC, BSHA. All authors read and approved the final manuscript.
